# Factors associated with alcohol screening and brief interventions: a cross-sectional study of cardiology clinicians in Sweden

**DOI:** 10.1186/s13722-025-00628-0

**Published:** 2025-11-27

**Authors:** Paul Welfordsson, Anna-Karin Danielsson, Anette Søgaard Nielsen, Caroline Björck, Bartosz Grzymala-Lubanski, Kristina Hambraeus, Olga Nilsson, Ida Haugen Löfman, Matthias Lidin, Sara Wallhed Finn

**Affiliations:** 1 Department of Global Public Health, Karolinska Institutet, Solna, 113 65 Sweden; 2 Unit of Clinical Alcohol Research, Institute of Clinical Research, University of Southern Denmark, Odense, Denmark; 3 Department of Women’s and Children’s Health, Akademiska Sjukhuset, Uppsala University, Uppsala, Sweden; 4 Centre for Research and Development, Region Gävleborg, Gävle, Sweden; 5 Department of Public Health and Clinical Medicine, Umeå University, Umeå, Sweden; 6 Department of Cardiology, Falun Hospital, Falun, Dalarna Sweden; 7 Department of Molecular Medicine and Surgery, Karolinska Institutet, Stockholm, Sweden; 8 Department of Vascular Surgery, Karolinska University Hospital, Stockholm, Sweden; 9 Department of Medicine, Unit of Cardiology, Karolinska Institutet, Stockholm, Sweden; 10 Department of Cardiology, Heart and Vascular Center, Karolinska University Hospital, Stockholm, Sweden; 11 Centre for Dependency Disorders, Stockholm, Sweden

**Keywords:** Cardiology, Alcohol, Cross-sectional survey, Stigma, Screening, Brief interventions, Self-efficacy

## Abstract

**Background:**

Alcohol screening and brief interventions (SBI) are effective strategies to reduce hazardous alcohol use in healthcare settings but are implemented inconsistently in cardiology practice. There is a need to understand factors associated with alcohol prevention practices in clinical cardiology to bridge this evidence-practice gap. The aim of this study was to investigate factors associated with SBI practices in cardiology.

**Methods:**

Multi-centre cross-sectional study. We surveyed clinicians at cardiology services in 12 regions across Sweden. The outcome was participants’ tendency to initiate SBI. Predictor variables included perceived importance of alcohol screening, level of comfort discussing alcohol habits with patients, perceived reliability of self-reported alcohol habits, and perceived competence for screening and brief interventions. Analyses included Wilcoxon signed-rank tests and ordinal logistic regression models.

**Results:**

In total, 692 clinicians participated in the survey (nurses = 55%; doctors = 19%; assistant nurses = 22%). Perceived importance of screening was not significantly associated with initiating SBI (OR = 1.55, 95%CI = 0.75–3.20). However, greater comfort when discussing alcohol habits was strongly associated with participants’ tendency to initiate SBI (OR = 4.06, 95%CI = 2.62–6.30) in maximally-adjusted models. Competence with screening (OR = 1.65, 95%CI = 1.06–2.56) and brief interventions (OR = 1.93, 95%CI = 1.30–2.85), and perceived reliability of self-reported alcohol habits (OR = 1.54, 95%CI = 1.12–2.13) were also positively associated with initiating SBI. While most (> 95%) participants considered it important that cardiology patients are asked about their alcohol habits, just 27% indicated that they often or always initiate SBI (*z* = 21.88, *p* < .001).

**Conclusions:**

Many cardiology clinicians in Sweden view alcohol screening as important, but these views are frequently not aligned with self-reported clinical practice. Findings highlight a need to empower clinicians to initiate conversations about alcohol use with their patients and for improved training to support SBI implementation. Promising strategies may include creating workflows that normalize discussions around alcohol use and clinician training that focuses on challenging stereotypes associated with alcohol use disorder.

**Supplementary Information:**

The online version contains supplementary material available at 10.1186/s13722-025-00628-0.

## Introduction

Alcohol use is a major global public health challenge, particularly in Europe where annual per-capita consumption of 9.8 L of pure alcohol ranks as the world’s highest [1]. Excessive consumption of alcohol is known to increase the risk of cardiovascular disease (CVD) [1, 2], cancers, and several other diseases [1], yet there has historically been little emphasis on alcohol prevention in cardiology services. One possible reason is reports of a ‘J-shaped curve’ in observational studies that purport to show reduced CVD mortality among regular, low-level drinkers [3, 4]. Such reports are controversial and are contradicted by newer Mendelian randomization studies [5, 6], yet may have contributed to mixed messages about alcohol use in clinical cardiology [7].

In light of accumulating evidence that even small quantities of alcohol can be harmful [1, 8], alcohol use is increasingly recognized as a modifiable risk factor [9]. Since 2021, European cardiology guidelines have stated that clinicians should ask about alcohol use in all medical evaluations and should support patients to moderate their alcohol consumption [10]. National healthcare guidelines in Sweden also recommend that patients should be screened for hazardous alcohol use – a quantity and frequency of consumption that significantly increases the risk of alcohol-related harm [11] – and encourage that patients with hazardous drinking should be offered brief interventions (BIs) [12]. These are low-intensity interventions [13] of approximately 10–15 min duration that typically involve motivational interviewing and advice on cutting down [14, 15].

Despite a growing body of evidence demonstrating effectiveness in hospital settings [16, 17], implementation of BIs in cardiology settings has been limited. In a recent survey of cardiology patients in Sweden, we found that about half of participants had been asked about alcohol use by staff [18]. Of these, just one in eight received a BI – despite up to 26% of the same patients reporting consumption consistent with hazardous use [19]. This evidence-practice gap is consistent with findings from other settings, which suggest that uptake of BIs has generally been slow [20, 21], and that alcohol screening and brief interventions (SBI) remain poorly integrated into healthcare practices [22–24]. Interestingly, there appears to be dissonance between cardiology clinicians’ stated views on the importance of SBI and the degree to which they engage in alcohol prevention practices [25, 26]; a survey in Sweden reported that cardiology staff consider it important to counsel their patients on unhealthy lifestyle habits – including alcohol use – yet do not prioritise this within their own practice [25].

While organizational factors such as excessive workload are well-documented barriers to SBI in general [25, 27–29], our recent qualitative study suggested that perceived stigma may be an important obstacle in the cardiology context [30]. Clinicians described alcohol as a sensitive topic and reported feeling uneasy discussing alcohol use with their patients. Clinicians perceived alcohol use to be underreported and some said they avoided the topic with patients due to concerns about triggering feelings of shame or harming the clinician-patient relationship. Indeed, alcohol use disorders are highly stigmatized [31] – a multi-step process that involves labeling, stereotyping and discrimination [32–34], and which introduces social distance between people [35] and delays treatment seeking [36]. Several studies have identified stigma as a barrier to SBI [37–40], partly by provoking negative emotions in clinicians during conversations about alcohol [41, 42]. In addition to triggering discomfort during clinical encounters, stigma may drive under-reporting of alcohol consumption and amplify clinicians’ concerns about the trustworthiness of self-reported alcohol use [30]. While comparisons of self-reported alcohol consumption with sales of alcoholic beverages suggest significant under-reporting [43, 44], this phenomenon is not specific to alcohol use, and also affects other lifestyle habits [45, 46]. Nevertheless, primary care-based studies indicate that patients are screened less frequently for alcohol use than for smoking, physical inactivity, and unhealthy diet, suggesting that clinicians are more reluctant to discuss alcohol consumption than other unhealthy habits [47, 48]. There is a need to understand factors associated with different lifestyle habits to develop effective implementation strategies.

Lack of training is widely-reported to be a key barrier to SBI [27, 28], and was identified during interviews with cardiology clinicians in our recent qualitative study [30]. Clinicians reported a low level of competence with BIs and described feeling powerless to address alcohol-related problems in their clinical work. To strengthen SBI implementation, additional investigation into clinicians’ perceived competence with alcohol prevention activities and factors associated with participation in SBI is thus warranted.

Ajzen’s theory of planned behaviour (TPB) emerged as an amalgamation of previous models and has since been established as a key basis for behavioural change studies [49]. The TPB asserts that individual attitudes and subjective norms shape peoples’ intentions and influence subsequent behaviours, for example their engagement in SBI. In the context of this study, individual attitudes may include clinicians’ views on the importance of alcohol screening or on the trustworthiness of self-reported lifestyle habits, while subjective norms may include perceived stigma around alcohol use. The TPB also includes the concept of perceived behavioural control, which builds upon and is closely related to Bandura’s theory of self-efficacy [50, 51], and which acts as an intrinsic motivator to engage in a specific behaviour, such as alcohol screening [27, 51]. According to Bandura’s original description of self-efficacy theory, negative emotions during conversations about alcohol use may interact with and further undermine clinicians’ self-efficacy for SBI [51]. However, these associations have yet to be investigated among cardiology staff.

The aim of this study was to investigate factors associated with SBI practices in cardiology. Research questions were as follows:

1. Which factors are associated with clinicians’ tendency to initiate SBI?
2. To what extent are cardiology clinicians’ views on the importance of alcohol screening consistent with their self-reported screening practices?
3. To what degree do clinicians feel comfortable discussing the following topics with patients: (a) alcohol use, (b) physical activity, (c) diet, and (d) smoking?
4. To what degree do clinicians perceive patients’ self-reporting of the following habits as reliable: (a) alcohol use, (b) physical activity, (c) diet, and (d) smoking?

## Methods

### Study design and ethical considerations

The study design was cross-sectional. We adhered to the STROBE statement (S1 Checklist) [52] and a preregistered study protocol and data analysis plan [53]. The study was conducted according to the Declaration of Helsinki. We obtained a formal ethical waiver from the Swedish Ethical Review Authority (2021-06819-01). All participants provided written informed consent by checking a box in the electronic questionnaire, in accordance with our ethical agreement.

### Setting

This was a multi-site study of hospital-based cardiology services in 12 administrative regions across Sweden: Dalarna, Gävleborg, Norrbotten, Skåne, Stockholm, Uppsala, Värmland, Västerbotten, Västra Götaland, Västmanland, Örebro, and Östergötland. Hospitals were selected to account for variation in regional factors that may affect healthcare practices, such as hospital type (university, district general) and catchment area.

### Participants and procedures

We included clinical staff (doctors, nurses, assistant nurses, and allied health professionals) working in cardiology units in Sweden. First, we contacted unit managers (by email or telephone) and provided information about the study. Managers who did not respond immediately were contacted up to three times in total. We asked managers who responded to forward a standardized recruitment email to all clinical staff working at their cardiology unit – including both inpatient and outpatient services. This email contained participant information and a link to a web questionnaire. Managers were asked to send two additional, standardized weekly reminder emails to the same staff. To calculate survey response rates, we asked managers to provide details of how many clinicians were included in their staff email lists. No participant incentives were offered.

### Data sources and measurement

We developed a novel questionnaire (S2 staff survey) to assess the variables described below, using REDCap electronic data capture tools hosted at Karolinska Institutet, Stockholm [54]. The questionnaire explored factors associated with the implementation of alcohol interventions in cardiology and included both check-boxes and optional free-text fields. The questionnaire underwent pilot testing with clinicians in November 2023 and took ≤ 5 min to complete. Survey data was collected between 1st January 2024–30th November 2024.

### Outcome variable

**Initiating SBI**: Participants’ tendency to initiate SBI was assessed as a categorical variable by asking, “How often do you initiate conversations about alcohol use with your patients?”. Responses options included: ’never’, ’rarely, with a few patients’, ’sometimes (about half of the time)’, ‘often, with most patients’, or ‘always, with every patient’. These responses were coded as 0–4, respectively.

### Predictor variables

Participants were asked to indicate the extent to which they agreed/disagreed with the following statements. Responses were recorded on five-point Likert scales, which were coded as: ‘strongly disagree’= 0; ‘disagree’= 1; ‘neither agree nor disagree’= 2; ‘agree’= 3; ‘strongly agree’= 4:

**Perceived importance of screening**: “I think it is important that cardiology patients are asked about their alcohol habits”. Factors related to perceived stigma, including **comfort discussing alcohol habits** (the degree to which alcohol was perceived as being a sensitive topic during clinical conversations): “I feel comfortable discussing alcohol habits with patients”, and **perceived reliability of self-reported alcohol habits**: “Patients’ self-reporting of alcohol habits is reliable”. Factors related to self-efficacy, including **perceived competence for screening**: “I feel sufficiently competent to ask patients about their alcohol habits”, and **perceived competence for BIs**; “I feel sufficiently competent to deliver BIs to patients”.

Other variables of interest included **comfort discussing physical activity**: “I feel comfortable discussing physical activity habits with patients”; **comfort discussing diet**: “I feel comfortable discussing dietary habits with patients”; **comfort discussing smoking**: “I feel comfortable discussing smoking habits with patients”; **perceived reliability of self-reported physical activity**: “Patients’ self-reporting of physical activity habits is reliable”; **perceived reliability of self-reported dietary habits**: “Patients’ self-reporting of dietary habits is reliable”, and **perceived reliability of self-reported smoking**: “Patients’ self-reporting of smoking habits is reliable”.

### Covariates

Participants self-reported their sociodemographic characteristics, including **gender** (man, woman, or other) and **age** (18–29, 30–39, 40–49, 50–59, ≥ 60 years). **Hospital type** was categorized as district general or university (S3 Table). Participants self-reported their professional background, including: **profession** (doctor, nurse, assistant nurse, other); years of **experience** post-qualification (‘≤3 years’, ‘4–10 years’, ‘≥10 years’), **specialist experience** (‘ischaemic heart disease’, ‘arrhythmia’, ‘heart failure’, ‘other’, ‘no specialist experience’), and cardiology **setting** (‘inpatients’ = all clinical cardiology staff who did not work in outpatients or in lab/intervention settings; ‘outpatients’ = all staff who worked in outpatient services, except those who also worked in lab/intervention settings; ‘lab/intervention unit’ = all staff who worked in the intervention unit, regardless of whether they also worked in another setting).

### Bias

To reduce selection and social desirability bias, information provided in the recruitment email emphasized that participation was anonymous, voluntary, and confidential, and that managers would remain unaware of clinicians’ willingness to participate, and of their survey responses. No payment or other incentives were offered.

### Study size

Since it was not feasible to perform meaningful sample size calculations using our bespoke survey instrument and sampling strategy, study size was pragmatic. We aimed to ensure adequate representation across Sweden’s diverse geographical regions and, after initially aiming to recruit 300 participants, we decided to contact additional cardiology units to increase geographical variation among participating cardiology services.

### Statistical analyses

Survey response rates were calculated by study site. We calculated medians with inter-quartile ranges for predictor and outcome variables, according to participant characteristics, and examined these for (independent) differences using Kruskal-Wallis tests. We examined associations between predictor variables and the study outcome using ordinal logistic regression. Predictors were included as binary variables in regression models. Responses ’strongly agree’ or ‘agree’ were coded as agree, and responses ‘neither agree nor disagree’, ‘disagree’, or ‘strongly disagree’ were coded as don’t agree. We applied stepwise adjustments for covariates that were significantly associated with the study outcome in univariate models. Interaction terms were examined for **comfort discussing alcohol habits**,** perceived competence for screening**, and **perceived competence for BIs**.

We calculated frequencies and percentages by response category for predictor and outcome variables and examined these for (within-subject) differences using Wilcoxon signed-rank tests. Bivariate correlations were expressed as Spearman’s rank order coefficients. Frequencies and percentages were then calculated by response category for clinicians’ comfort discussing lifestyle habits and the perceived reliability of self-reported lifestyle habits; we examined for (within-subject) differences in the distributions of responses across the four lifestyle habits using Friedman tests. Where statistically significant differences were found, we computed post-hoc pairwise comparisons using Wilcoxon signed-rank tests and applied the Bonferroni correction for multiple comparisons (adjusted *p*-value = 0.0083). Results were reported as odds ratios (ORs) with 95% confidence intervals (CIs) and *p*-values < 0.05 were considered statistically significant. Statistical analyses were conducted in StataSE v17.

### Free-text response analysis methods

In addition to the statistical methods described above, we used deductive content analysis to identify free-text responses that related to stigma, self-efficacy, or the perceived importance of SBI. Findings were reported as a brief narrative summary with supporting quotations.

## Results

### Participants

**Fig. 1:**
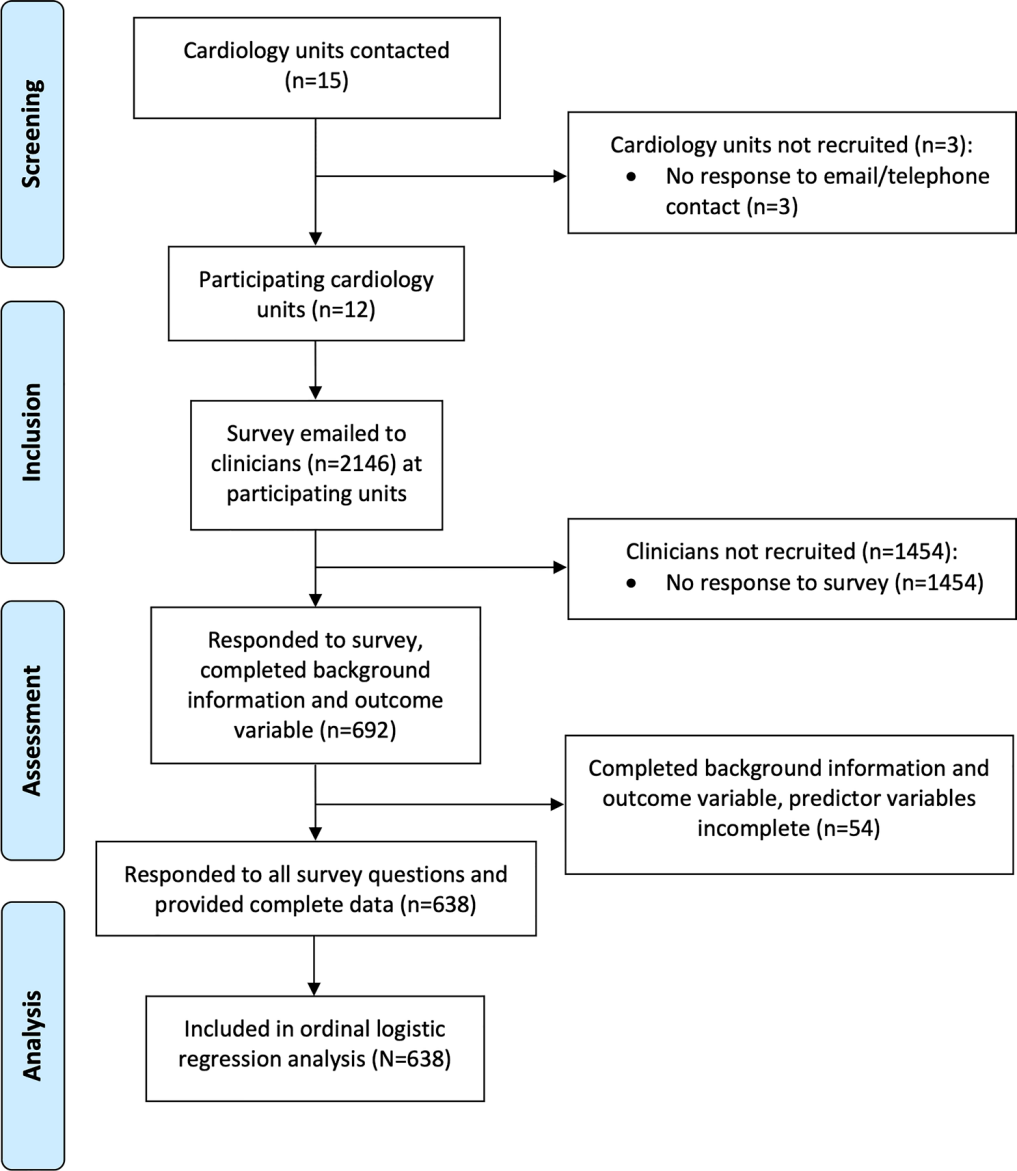
Flow of participants through the study

A total of 2146 cardiology clinicians were contacted by unit managers, of whom 692 (32.2%) responded to the survey. Response rates are reported by study site in Supplementary material 3. Almost half of participants worked in Sweden’s two largest cities, Stockholm (29%) and Gothenburg (15%), while the rest were spread across the country. Mean age was 45.0 years (SD = 12.3, range = 19–74 years) and 76% of the participants were women. Most participants (55%) were nurses, while doctors and assistant nurses each comprised about 20%. Half of the participants reported having 10 years of work experience or more. Almost half (46%) worked in inpatient settings, while about a third worked in outpatients and the rest worked in lab/intervention settings. Just under half of participants (47%) reported having specialist cardiology experience (Table 1). Flow of participants through the study is reported in Fig. 1.

**Table 1:** Predictor variables and study outcome, initiating SBI, according to participant characteristics (*N* = 692)

**Characteristic**	**Total (%)**	Predictor variables										Outcome variable	
		Attitude		Stigma-related factors				Self-efficacy-related factors					
		**Perceived importance of screening** ^**a**^		**Comfort discussing alcohol habits** ^**b**^		**Perceived reliability of self-reported alcohol habits** ^**c**^		**Perceived competence for screening** ^**d**^		**Perceived competence for brief interventions** ^**e**^		**Initiating SBI**	
		**Median (IQR)**	***p*** **-value**	**Median (IQR)**	***p*** **-value**	**Median (IQR)**	***p*** **-value**	**Median (IQR)**	***p*** **-value**	**Median (IQR)**	***p*** **-value**	**Median (IQR)**	***p*** **-value**
** All participants: **	692 (100)	4 (4–4)		3 (2–4)		2 (1–3)		3 (2–4)		2 (1–3)		1 (1–3)	
** Gender: **			0.075		0.059		0.054		**0.001**		**0.001**		**0.022**
Woman	525 (75.9)	4 (4–4)		3 (2–4)		2 (1–3)		3 (2–3)		2 (1–3)		1 (1–3)	
Man	164 (23.7)	4 (3–4)		3 (2–4)		1 (1–3)		3 (2–4)		3 (1–3)		2 (1–3)	
Other	3 (0.4)	4 (3–4)		4 (2–4)		1 (1–3)		4 (3–4)		3 (2–4)		2 (0–4)	
** Age group (years): **			0.972		**0.001**		0.118		**< 0.001**		**< 0.001**		**0.023**
18–29	86 (12.2)	4 (4–4)		3 (2–3)		2 (1–3)		3 (1–3)		1 (1–2)		1 (0–2)	
30–39	162 (23.4)	4 (4–4)		3 (2–4)		1 (1–3)		3 (1–3)		2 (1–3)		1 (1–3)	
40–49	170 (24.6)	4 (4–4)		3 (3–4)		2 (1–3)		3 (3–4)		3 (1–3)		1 (1–3)	
50–59	174 (25.1)	4 (4–4)		3 (2–4)		2 (1–3)		3 (2–4)		2 (1–3)		1 (1–3)	
≥ 60	100 (14.5)	4 (4–4)		3 (2–4)		2 (1–3)		3 (2–4)		2 (1–3)		1 (1–3)	
** Hospital type: **			0.295		0.351		**0.008**		0.758		0.664		0.275
District general	169 (24.4)	4 (4–4)		3 (2–3)		1 (1–3)		3 (2–4)		2 (1–3)		1 (1–3)	
University	523 (75.6)	4 (4–4)		3 (2–4)		2 (1–3)		3 (2–3)		2 (1–3)		1 (1–3)	
** Profession: **			0.776		**< 0.001**		0.618		**< 0.001**		**< 0.001**		**< 0.001**
Assistant nurse	150 (21.7)	4 (4–4)		3 (1–3)		2 (1–3)		2 (1–3)		1 (0–2)		1 (0–1)	
Nurse	379 (54.8)	4 (4–4)		3 (2–4)		2 (1–3)		3 (2–3)		2 (1–3)		1 (1–3)	
Doctor	128 (18.5)	4 (4–4)		4 (3–4)		2 (1–3)		4 (3–4)		3 (3–4)		2 (2–3)	
Other profession	35 (5.1)	4 (3–4)		3 (2–4)		2 (1–3)		3 (1–3)		1 (0–3)		1 (0–1)	
** Experience level: **			0.412		**0.014**		0.893		**< 0.001**		**< 0.001**		**< 0.001**
≤ 3 years	158 (22.8)	4 (4–4)		3 (2–3)		2 (1–3)		3 (1–3)		2 (1–3)		1 (0–2)	
4–10 years	183 (26.5)	4 (4–4)		3 (2–4)		2 (1–3)		3 (2–3)		2 (1–3)		1 (1–3)	
≥ 10 years	351 (50.7)	4 (4–4)		3 (2–4)		2 (1–3)		3 (2–4)		3 (1–3)		1 (1–3)	
** Cardiology setting: **			0.127		**< 0.001**		0.855		**< 0.001**		**< 0.001**		**< 0.001**
Inpatients	317 (45.8)	4 (3–4)		3 (2–3)		2 (1–3)		3 (1–3)		2 (1–3)		1 (0–2)	
Outpatients	250 (36.1)	4 (4–4)		3 (3–4)		2 (1–3)		3 (3–4)		3 (2–3)		2 (1–3)	
Lab/intervention	125 (18.1)	4 (4–4)		3 (2–4)		2 (1–3)		3 (2–4)		2 (1–3)		1 (1–2)	
** Specialist experience: **			0.362		**< 0.001**		0.364		**< 0.001**		**< 0.001**		**< 0.001**
None	372 (53.8)	4 (4–4)		3 (2–3)		2 (1–3)		3 (1–3)		2 (1–3)		1 (0–2)	
IHD	101 (14.6)	4 (4–4)		3 (3–4)		2 (1–3)		3 (2–4)		3 (1–3)		2 (1–3)	
Arrhythmia	72 (10.4)	4 (4–4)		3 (2–4)		2 (1–3)		3 (2–4)		3 (2–3)		2 (1–3)	
Heart failure	88 (12.7)	4 (4–4)		3 (3–4)		2 (1–3)		3 (3–4)		3 (2–3)		3 (1–3)	
Other specialist	59 (8.5)	4 (3–4)		3 (2–4)		2 (1–3)		3 (2–4)		3 (1–3)		1 (1–2)	

*P-*values refer to Kruskal-Wallis tests

a)* n* = 650, b) *n* = 642, c) *n* = 638, d) *n* = 650, e) *n* = 650

Legend: Predictor variables (all), strongly disagree’ = 0, ‘disagree’ = 1, ‘neither agree nor disagree’ = 2, ‘agree’ = 3, ‘strongly agree’ = 4, Outcome variable (Initiating SBI), ’never’=0, ’rarely, with a few patients’=1, ’sometimes (about half of the time)’=2, ‘often, with most patients’=3, ‘always, with every patient’=4

### Factors associated with initiating SBI

Ordinal logistic regression analyses indicated statistically significant univariate associations between predictor variables and participants’ tendency to initiate SBI (Table 2). After adjusting for other predictor variables (Model 1), perceived importance of screening was no longer significantly associated with initiating SBI (OR = 1.55, 95%CI = 0.75–3.20), and was thus dropped from subsequent models. In Model 4, our maximally-adjusted model, greater comfort when discussing alcohol habits remained strongly associated with participants’ tendency to initiate SBI (OR = 4.06, 95%CI = 2.62–6.30). Competence with screening and BIs were also associated with initiating SBI, with odds ratios of 1.65 (95%CI = 1.06–2.56) and 1.93 (95%CI = 1.30–2.85), respectively. The odds ratio for perceived reliability of self-reported alcohol habits was slightly lower than for other predictors, but its association with initiating SBI remained statistically significant (OR = 1.54, 95%CI = 1.12–2.13).

**Table 2:** Association between predictor variables and initiating SBI; ordinal logistic regression models (*N* = 638)

	OR (95% CI)				
	Univariate	Model 1	Model 2	Model 3	Model 4
**Importance of screening:**					
Don’t agree	Ref	Ref			
Agree	**2.63 (1.39–4.95)**	1.55 (0.75–3.20)			
**Comfortable discussing alcohol:**					
Don’t agree	Ref	Ref	Ref	Ref	Ref
Agree	**8.59 (6.01–12.27)**	**3.61 (2.38–5.48)**	**4.01 (2.64–6.10)**	**4.13 (2.68–6.37)**	**4.06 (2.62–6.30)**
**Reliability of self-reported alcohol:**					
Don’t agree	Ref	Ref	Ref	Ref	Ref
Agree	**1.80 (1.33–2.44)**	**1.37 (1.00–1.87)**	**1.42 (1.04–1.95)**	**1.51 (1.10–2.09)**	**1.54 (1.12–2.13)**
**Competence with screening:**					
Don’t agree	Ref	Ref	Ref	Ref	Ref
Agree	**6.43 (4.63–8.94)**	**2.02 (1.34–3.06)**	**1.98 (1.30–3.01)**	**1.76 (1.14–2.73)**	**1.65 (1.06–2.56)**
**Competence with BIs:**					
Don’t agree	Ref	Ref	Ref	Ref	Ref
Agree	**5.87 (4.30–8.00)**	**2.46 (1.70–3.56)**	**2.47 (1.70–3.59)**	**2.14 (1.45–3.15)**	**1.93 (1.30–2.85)**

Model 1 includes predictor variables (perceived importance of screening, comfort discussing alcohol habits, perceived reliability of self-reported alcohol habits, perceived competence for screening, perceived competence for BIs)

Model 2 includes predictor variables and demographic characteristics (gender, age group)

Model 3 includes predictor variables, demographic characteristics, and professional characteristics (profession, experience level, specialist experience)

Model 4 includes predictor variables, demographic characteristics, professional characteristics, and cardiology setting

Gender = ’other’ excluded from adjusted models 1–4 (*n* = 635)

Interaction terms were added to explore whether associations between perceived competence with alcohol screening and initiating SBI, or between perceived competence with BIs and initiating SBI, were moderated by clinicians’ comfort levels when discussing alcohol habits. These interaction terms were not statistically significant and did not improve model fit (likelihood-ratio test: *p* = .587), suggesting that the association between self-efficacy for SBI (either for screening or BIs) and clinicians’ tendency to initiate conversations about alcohol use does not vary according to their level of comfort when discussing alcohol habits with patients.

### Perceived importance of alcohol screening

**Fig. 2:**
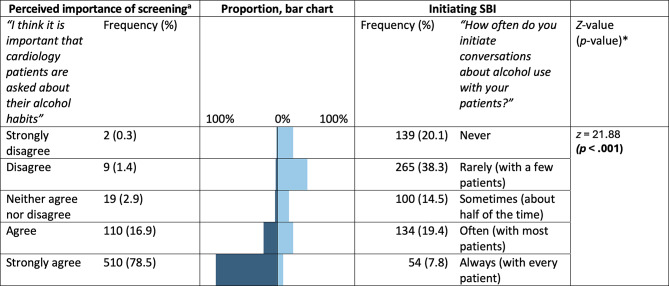
Response categories for perceived importance of screening and outcome variable, initiating SBI; frequencies and percentages (*N* = 692). (a) n = 650. *Refer to Wilcoxon signed-rank test

Over 95% of participants either agreed or strongly agreed that it is important that cardiology patients are asked about their alcohol habits (median response = strongly agree). However, just 27% of participants indicated that they either often or always initiate conversations about alcohol use with their patients (median response = rarely, with a few patients). Overall, there was a significant difference between the stated importance of alcohol screening and clinicians’ self-reported screening practices, with higher perceived importance for screening and lower tendency to initiate SBI (*z* = 21.88, *p* < .001, Fig. 2). The correlation between perceived importance of screening and initiating SBI was 0.218 (*p* < .001, Fig. S4).

### Comfort when discussing alcohol and other lifestyle habits

**Fig. 3:**
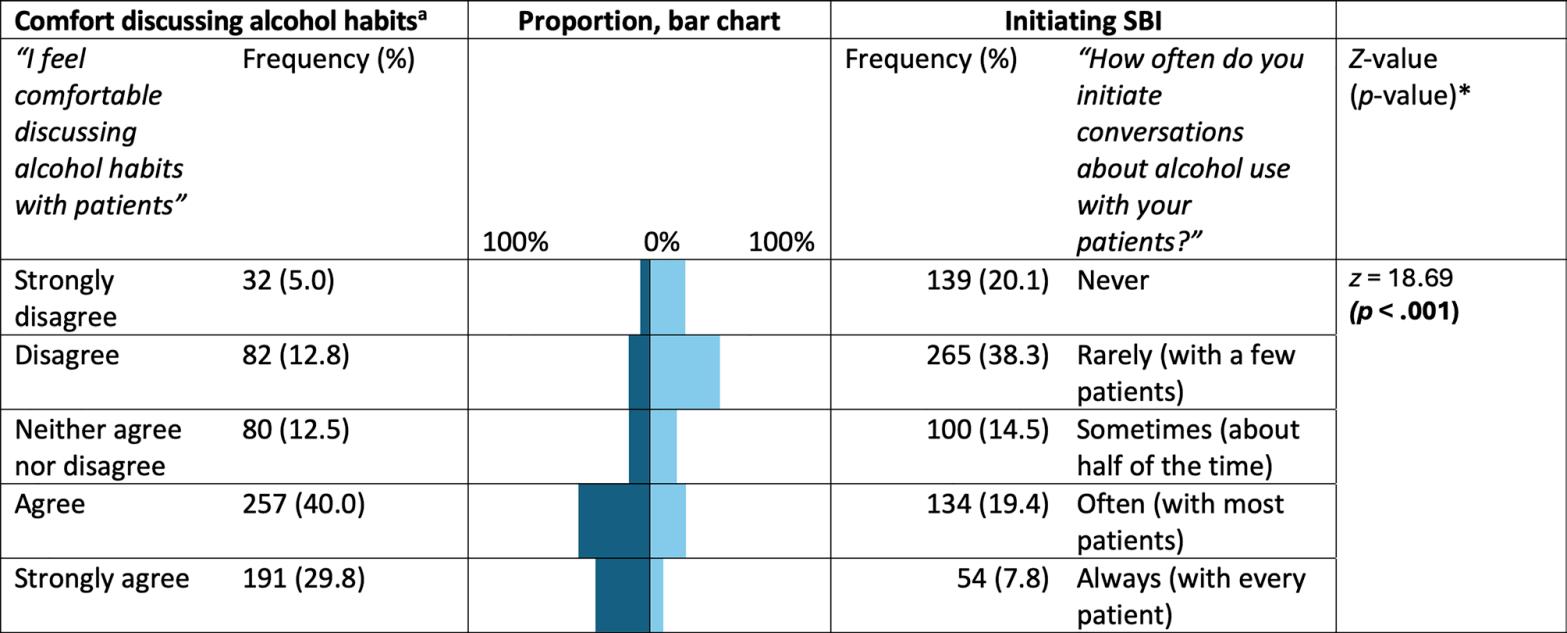
Response categories for comfort discussing alcohol habits and outcome variable, initiating SBI; frequencies and percentages (*N* = 692). (a) n = 642. *Refer to Wilcoxon signed-rank test

Clinicians’ reported comfort levels varied significantly across the four lifestyle habits studied (*p* < .001, Table 3). On average, lower levels of comfort were reported when discussing alcohol habits and diet (median response = agree) than physical activity and smoking (median response = strongly agree). Just under 70% of participants reporting feeling comfortable discussing alcohol habits with patients (agree or strongly agree), compared to 84% for diet, 88% for physical activity, and 89% for smoking habits. Post-hoc pairwise comparisons indicated that clinicians were significantly less comfortable (Bonferroni corrected *p* < .0083) when discussing alcohol habits with patients than either smoking (*z *= -15.60, *p* < .001), physical activity (*z *= -14.94, *p* < .001), or diet (*z *= -11.69, *p*  < .001). At the same time, clinicians were significantly less comfortable discussing diet with patients than smoking (*z *= -7.11, *p* < .001) or physical activity (*z *= -6.69, *p* < .001). There was no significant difference between clinicians’ reported comfort levels when discussing physical activity and those when discussing smoking (*z *= -1.37, *p* = .170). There was, however, a significant difference between clinicians’ reported level of comfort discussing alcohol habits and their self-reported screening practices (*z* = 18.69, *p* < .001, Fig. 3). The correlation between comfort discussing alcohol habits and initiating SBI was 0.551 (*p* < .001, Fig. S5).

**Table 3:** Clinicians’ reported comfort in discussing alcohol habits, physical activity, diet, and smoking habits (*N* = 642)

	“I feel comfortable discussing […] with patients”, frequency (%)				
Response:	Alcohol habits	Physical activity	Diet	Smoking habits	*P*-value
Strongly disagree	32 (5.0)	5 (0.8)	7 (1.1)	7 (1.1)	**< 0.001**
Disagree	82 (12.8)	24 (3.8)	26 (4.0)	26 (4.0)	
Neither agree nor disagree	80 (12.5)	45 (7.0)	69 (10.8)	35 (5.5)	
Agree	257 (40.0)	207 (32.2)	249 (38.8)	201 (31.3)	
Strongly agree	191 (29.7)	361 (56.2)	291 (45.3)	373 (58.1)	

*P-*value refers to Friedman test

### Perceived reliability of self-reported lifestyle habits

Clinicians’ perceptions of the reliability of self-report also varied significantly across the lifestyle habits studied (*p* < .001, Table 4). On average, clinicians perceived self-reported smoking habits to be most reliable (median response = agree), followed by alcohol use, physical activity, and dietary habits (median response = neither agree nor disagree). Just under 33% of participants perceived patients’ self-reported alcohol habits to be reliable (agree or strongly agree), compared to 49% for diet and physical activity, and 59% for smoking habits. Post-hoc pairwise comparisons (Bonferroni corrected *p* < .0083) indicated the following differences: Clinicians perceived self-reported alcohol habits to be significantly less reliable that self-reported smoking (*z *= -13.53, *p* < .001), physical activity (*z *= -11.17, *p* < .001), or dietary habits (*z*=-11.02, *p* < .001). At the same time, clinicians perceived lower reliability for self-reported dietary habits than smoking habits (*z *= -5.46, *p* < .001), and lower reliability for self-reported physical activity than smoking (*z *= -5.29, *p* < .001). There was no significant difference between the perceived reliability of self-reported physical activity and that for dietary habits (*z* = 0.13, *p* = .895). There was, however, a significant difference between clinicians’ perceptions of the reliability of self-reported alcohol habits and their self-reported alcohol screening practices (*z* = 4.08, *p* < .001, Fig. S6). The correlation between perceived reliability of self-reported alcohol habits and initiating SBI was 0.156 (*p* < .001, Fig. S7).

**Table 4:** Perceived reliability of self-reported alcohol habits, physical activity, dietary habits, and smoking habits (*N* = 638)

	“Patients’ self-reporting of […] is reliable”, frequency (%)				
Response:	Alcohol habits	Physical activity	Diet	Smoking habits	*P*-value
Strongly disagree	32 (5.0)	9 (1.4)	11 (1.7)	10 (1.6)	**< 0.001**
Disagree	257 (40.3)	183 (28.7)	180 (28.2)	133 (20.8)	
Neither agree nor disagree	140 (21.9)	134 (21.0)	134 (21.0)	117 (18.3)	
Agree	200 (31.4)	277 (43.4)	276 (43.3)	317 (49.7)	
Strongly agree	9 (1.4)	35 (5.5)	37 (5.8)	61 (9.6)	

*P-*value refers to Friedman test

### Perceived competence with SBI

Overall, 64% of participants indicated (agreed or strongly agreed; median response = agree) that they felt sufficiently competent to ask patients about their alcohol habits, while 44% indicated (agreed or strongly agreed; median response = neither agree nor disagree) that they felt competent to deliver BIs. We found significant differences between clinicians’ perceived competence for alcohol screening and their self-reported screening practices (*z* = 16.82, *p* < .001, Fig. S8), and between clinicians’ perceived competence for BIs and their self-reported screening practices (*z* = 7.17, *p* < .001, Fig. S9). The correlation between perceived competence with alcohol screening and initiating SBI was 0.512 (*p* < .001, Fig. S10). The respective correlation between perceived competence with BIs and initiating SBI was 0.521 (*p* < .001, Fig. S11).

### Free text responses

In total, 29 survey respondents provided free-text responses to the question, ‘do you have any other comments?’. Of these, 19 addressed issues of perceived stigma surrounding unhealthy lifestyle habits – particularly alcohol use. One respondent noted that, “alcohol use is more stigmatized than tobacco use, and is more prone to underreporting”, while another remarked that, “I think it’s rude to talk about alcohol, I don’t want to ask. It’s their privacy – if they raise the issue you can answer”. Seventeen respondents made generalized statements about the reliability of self-reported lifestyle habits, including: “patients often tell a distorted picture of reality”, and “many patients exaggerate in the healthier direction”. Overall, free-text responses tended to express a distrust of self-reported lifestyle habits. One participant remarked that, “I always take self-report with a pinch of salt”, while another concluded that, “people with overconsumption and/or underactivity often live in shame and, not infrequently, in denial.”

Issues of self-efficacy were less widely discussed, emerging in six free-text responses. One respondent noted that, “I often talk about the importance of quitting smoking but I don’t feel like I have the same knowledge regarding the impact of alcohol on CVD”, while another commented that, “I would like to learn more about how to talk to patients about alcohol use in a successful way.”

## Discussion

This cross-sectional study of 692 cardiology clinicians found that participants’ views on the importance of alcohol screening were often inconsistent with their self-reported screening practices. Participants viewed alcohol screening as important yet were less comfortable discussing alcohol use than other unhealthy lifestyle habits. Self-reported alcohol use was viewed as less reliable than self-reported physical activity, diet, or smoking. Greater comfort, greater confidence in self-reported alcohol use, and greater perceived competence with alcohol prevention practices were associated with increased odds of initiating SBI. Findings highlight the need to empower clinicians to discuss alcohol use with their patients and suggest that strategies to strengthen self-efficacy may be important during SBI implementation.

Our findings support those of previous studies that have reported a disconnect between the stated importance of promoting healthy lifestyle habits and how clinicians engage in delivering lifestyle interventions [25, 26]. A 2021 study reported that 69%–94% of cardiology staff stated that it is important to counsel patients on unhealthy lifestyle habits, yet only 18%–41% screened their own patients’ lifestyle habits and 11%–23% provided counselling to patients [25]. That study was limited by a relatively small sample of 251 clinicians from two hospitals in Stockholm, yet our results suggest that this pattern may extend to cardiology staff across Sweden. Kromme et al. reported similar findings in a qualitative study of internal medicine physicians’ involvement in health promotion in the Netherlands [26]; participants regarded promoting a healthy lifestyle as important yet were ambivalent about their own role in health promotion. Physicians had negative views of patients with unhealthy lifestyle habits and expressed doubts about their own ability to motivate and effect change in these patients. Kromme et al. also identified time constraints as a barrier to health promotion practices, an observation consistent with evidence from the alcohol prevention literature [25, 27–29].

Our study points to a possible link between stigma-related factors and engagement in alcohol prevention practices in the cardiology context [26, 37–39]. Our finding that clinicians experience feelings of discomfort when discussing alcohol use is consistent with broader evidence from previous systematic reviews [41, 42]. Physicians may downplay discussions of alcohol use during consultations to avoid stigmatizing labels [30], and may avoid intervening in alcohol use out of concern for the clinician-patient relationship [26, 41]. Encouragingly though, studies suggest that such concerns may be unfounded; SBI is generally well-accepted by patients in hospital settings [55–58]. Our recent qualitative study suggested that clinicians may feel more comfortable discussing the issue of alcohol use when patients raise it themselves [30], highlighting that initiating such conversations can be a challenging first step.

The current study found that clinicians were less inclined to trust the reliability of patients’ self-reported alcohol use in comparison to other lifestyle habits – a novel finding in the cardiology context. Evidence suggests that alcohol consumption is underreported by up to 50% in Sweden [43, 44], yet under-reporting is also known to affect other lifestyle habits, and is likely to be context dependent [59]. Tobacco use, for example, has been shown to be underreported by up to 90% according to urinary nicotine levels among participants in smoking cessation trials [45], while comparisons of self-reported physical activity with accelerometer-based assessment methods also indicate inconsistencies [46]. It is not clear exactly what is driving clinicians’ specific concerns about the trustworthiness of self-reported alcohol use, but factors such as social desirability bias [60] and negative views of patients with alcohol use disorders may contribute [61]. However, concerns about the trustworthiness of self-reported alcohol use may also arise from perceived difficulties in quantifying alcohol use [26] and suspected recall bias [62].

Lack of training has been reported to be a key barrier to SBI in primary care [27] and inpatient hospital settings [28]. The current study suggests that perceived competence with both screening and with BIs are important in cardiology, even after adjusting for the clinical context and individual characteristics. This is consistent with findings from our 2024 qualitative study, which identified a shortfall in training among cardiology clinicians, who reported that they felt unqualified to address alcohol issues with their patients [30]. A limitation of that study was its sample size of 24 clinicians and unclear transferability to other cardiology services, yet the current report suggests that low self-efficacy may be a barrier to SBI for many clinicians in Sweden.

According to the TPB, clinicians’ attitudes, subjective norms, and perceived behavioural control can influence their engagement in alcohol prevention activities [49]. This study found that positive attitudes to the importance of screening were common among clinicians, yet were a rather weak predictor of initiating SBI. This is in keeping with findings from ODHIN – a large, multi-national study of SBI implementation that found no association between professional attitudes and engagement in SBI [63]. In contrast, we found that widely held views that alcohol use is under-reported, which may partly reflect negative attitudes, were associated with lower participation in SBI. At the same time, the TPB suggests that subjective norms surrounding alcohol use may influence clinicians’ screening behaviours. Perceived stigma may trigger negative emotions among clinicians when discussing alcohol use, undermining their propensity to engage in SBI [41, 42]. Such discomfort may be amplified by cognitive dissonance, a process in which psychological stress results from perceived inconsistencies between clinicians’ beliefs that alcohol screening is important and their experience of ambivalence towards initiating SBI [64]. Finally, perceived shortcomings in behavioural control, or low self-efficacy, may have weakened clinicians’ intrinsic motivation for SBI [51], thus reducing their tendency to initiate conversations about alcohol use [65]. While the TPB and self-efficacy theory suggest that experiencing negative emotions during conversations about alcohol use may reinforce clinicians’ low self-efficacy for SBI [51], we did not find evidence of such interaction effects, possibly due to measurement limitations and our cross-sectional study design.

Overall, our study suggests that work is needed to ensure that clinicians feel both comfortable and capable when discussing patients’ alcohol habits. Promising SBI implementation stategies may include measures to reduce public stigma surrounding alcohol use disorders and educational methods which help providers to understand the cognitions and emotions underlying self-reported alcohol consumption. The US-based National Institute on Alcohol Abuse and Alcoholism (NIAAA) recommends several patient-level and practice-level strategies to reduce perceived stigma associated with alcohol use disorders [66]. Patient-level recommendations include encouraging patient autonomy, using non-stigmatizing language, and educating patients about evidence-based treatment options [67]. Practice-level recommendations include creating workflows and systems that normalize discussions around alcohol use in healthcare settings [68], maximizing patient privacy, working collaboratively using an interdisciplinary approach, and providing education to the entire healthcare team [66]. Specific educational strategies may include increasing contact with people affected by alcohol use disorder during clinical training and addressing stereotypes through vignettes, case studies, and real-world examples [69–71]. Education should promote an awareness that the majority people with alcohol use disorder have low-to-moderate severity and experience few social problems [72], and that effective treatments are available. More broadly, training of healthcare staff should emphasize the high prevalence and broad spectrum of alcohol use, rather than reinforcing a false dichotomization of ‘normal’ and problem drinkers [73, 74].

### Strengths and limitations

Study strengths included a multi-centre design and a larger sample than previous studies, which allowed us to examine a broad range of factors associated with SBI. Our statistical approach was conservative and emphasized the ordinal nature of the variables studied. We adjusted for multiple comparisons where appropriate and applied multiple analytical approaches, including non-parametric tests, bivariate correlations, multivariate regression, and qualitative content analysis – demonstrating consistent findings. Limitations included a cross-sectional design, the lack of a formal validation study for our survey instrument, and a reliance on self-reported outcome data. While we assessed hospital type and clinical cardiology setting, the study focused mainly on individual predictors of engagement in SBI and did not consider contextual indications for SBI, such as when prescribing medications, nor system-level factors such as institutional policies. Our findings are from a Swedish context and may not be generalizable, however similar barriers to SBI have been identified in other contexts [28, 38].

## Conclusions

Many cardiology clinicians in Sweden view alcohol screening as important, but these views are often not aligned with self-reported clinical practice. Clinicians were generally less comfortable discussing alcohol use than other lifestyle habits and tended to view self-reported alcohol use as less reliable than self-reported physical activity, diet, or smoking. Higher levels of clinician comfort, perceived competence, and confidence in patients’ self-reported alcohol use were associated with a higher likelihood of engaging in SBI. Findings highlight a need to empower clinicians to initiate conversations about alcohol use with their patients and for improved training to support SBI implementation. Promising strategies may include creating workflows that normalize discussions around alcohol use and clinical training that focuses on challenging stereotypes associated with alcohol use disorder.

## Supplementary information

Below is the link to the electronic supplementary material.

Supplementary Material 1

## Data Availability

This study adhered to a preregistered, publicly available study protocol and data analysis plan [https://osf.io/4vy5h]. De-identified study data are available on reasonable request from sara.wallhed-finn@ki.se.
