# Supplementary material for: Factors associated with alcohol screening and brief interventions: a cross-sectional study of cardiology clinicians in Sweden
Source: Addict Sci Clin Pract. 2025 Nov 27;20:92. doi: 10.1186/s13722-025-00628-0 (PMC12665207; doi:10.1186/s13722-025-00628-0)
Supplement: Supplementary file 1 — Supplementary Material 1 [file 13722_2025_628_MOESM1_ESM.docx]

**SUPPLEMENTARY MATERIALS**

**S1** **Checklist:**

STROBE Statement—Checklist of items that should be included in reports of ***cross-sectional studies***

|  | Item No | Recommendation | Page number |
| --- | --- | --- | --- |
| **Title and abstract** | 1 | (*a*) Indicate the study’s design with a commonly used term in the title or the abstract | Title page |
|  |  | (*b*) Provide in the abstract an informative and balanced summary of what was done and what was found | Title page, 1-2 |
| Introduction | | |  |
| Background/rationale | 2 | Explain the scientific background and rationale for the investigation being reported | 3-6 |
| Objectives | 3 | State specific objectives, including any prespecified hypotheses | 6-7 |
| Methods | | |  |
| Study design | 4 | Present key elements of study design early in the paper | 7 |
| Setting | 5 | Describe the setting, locations, and relevant dates, including periods of recruitment, exposure, follow-up, and data collection | 7 |
| Participants | 6 | (*a*) Give the eligibility criteria, and the sources and methods of selection of participants | 8 |
| Variables | 7 | Clearly define all outcomes, exposures, predictors, potential confounders, and effect modifiers. Give diagnostic criteria, if applicable | 8-10 |
| Data sources/ measurement | 8* | For each variable of interest, give sources of data and details of methods of assessment (measurement). Describe comparability of assessment methods if there is more than one group | 8 |
| Bias | 9 | Describe any efforts to address potential sources of bias | 10-11 |
| Study size | 10 | Explain how the study size was arrived at | 11 |
| Quantitative variables | 11 | Explain how quantitative variables were handled in the analyses. If applicable, describe which groupings were chosen and why | 9-12 |
| Statistical methods | 12 | (*a*) Describe all statistical methods, including those used to control for confounding | 11-12 |
|  |  | (*b*) Describe any methods used to examine subgroups and interactions | N/A |
|  |  | (*c*) Explain how missing data were addressed | 11-12 |
|  |  | (*d*) If applicable, describe analytical methods taking account of sampling strategy | N/A |
|  |  | (*e*) Describe any sensitivity analyses | N/A |
| Results | | |  |
| Participants | 13* | (a) Report numbers of individuals at each stage of study—eg numbers potentially eligible, examined for eligibility, confirmed eligible, included in the study, completing follow-up, and analysed | 13, Fig. 1 |
|  |  | (b) Give reasons for non-participation at each stage | Fig. 1 |
|  |  | (c) Consider use of a flow diagram | Fig. 1 |
| Descriptive data | 14* | (a) Give characteristics of study participants (eg demographic, clinical, social) and information on exposures and potential confounders | 12-13, Table 1 |
|  |  | (b) Indicate number of participants with missing data for each variable of interest | Table 1, |
| Outcome data | 15* | Report numbers of outcome events or summary measures | Table 1, 13-19 |
| Main results | 16 | (*a*) Give unadjusted estimates and, if applicable, confounder-adjusted estimates and their precision (eg, 95% confidence interval). Make clear which confounders were adjusted for and why they were included | Tables 2-9 |
|  |  | (*b*) Report category boundaries when continuous variables were categorized | N/A |
|  |  | (*c*) If relevant, consider translating estimates of relative risk into absolute risk for a meaningful time period | N/A |
| Other analyses | 17 | Report other analyses done—eg analyses of subgroups and interactions, and sensitivity analyses | 19-21 |
| Discussion | | |  |
| Key results | 18 | Summarise key results with reference to study objectives | 21 |
| Limitations | 19 | Discuss limitations of the study, taking into account sources of potential bias or imprecision. Discuss both direction and magnitude of any potential bias | 25-26 |
| Interpretation | 20 | Give a cautious overall interpretation of results considering objectives, limitations, multiplicity of analyses, results from similar studies, and other relevant evidence | 21-26 |
| Generalisability | 21 | Discuss the generalisability (external validity) of the study results | 25 |
| Other information | | |  |
| Funding | 22 | Give the source of funding and the role of the funders for the present study and, if applicable, for the original study on which the present article is based | Title page |

*Give information separately for exposed and unexposed groups.

**Note:** An Explanation and Elaboration article discusses each checklist item and gives methodological background and published examples of transparent reporting. The STROBE checklist is best used in conjunction with this article (freely available on the Web sites of PLoS Medicine at http://www.plosmedicine.org/, Annals of Internal Medicine at http://www.annals.org/, and Epidemiology at http://www.epidem.com/). Information on the STROBE Initiative is available at [www.strobe-statement.org](http://www.strobe-statement.org).

**S2 Staff survey:**

**About you:**

1. Which region do you work in ?

- Stockholm
- Uppsala
- Gävleborg
- Dalarna
- Västra Götalandsregionen
- Skåne
- Norrbotten
- Östergötland
- Örebro län
- Västerbotten
- Sörmland
- Jönköpings län
- Kronoberg
- Kalmar län
- Gotland
- Blekinge
- Halland
- Värmland
- Västmanland
- Västernorrland
- Jämtland
- Härjedalen

1. How do you define your gender identity?

- Male
- Female
- Other

1. What is your age?

- ______ years

1. What is your profession?

- Doctor
- Nurse
- Assistant nurse
- Other (please specify)

1. How many years have you worked as a qualified practitioner?

- ≤3 years
- 4–10 years
- ­≥10 years

1. Which cardiology settings do you work in? (tick all that apply)

- Coronary care unit
- Ward
- Outpatients
- Lab/intervention unit
- Other (please specify)

1. Do you have any specialist experience?

- Ischaemic heart disease
- Arrhythmia
- Heart failure
- Other (please specify)
- No specialist experience

1. How often do you initiate conversations about alcohol use with your patients?

|  | - Never |
| --- | --- |
|  | - Rarely, with a few patients |
|  | - Sometimes (about half of the time) |
|  | - Often, with most patients |
|  | - Always, with every patient |

**Alcohol habits and alcohol problems**

Indicate how much you agree or disagree with the following statement regarding the patients that you work with:

1. I feel sufficiently competent to ask patients about their alcohol habits

- Strongly Disagree
- Disagree
- Neither Agree nor Disagree
- Agree
- Strongly Agree

1. I feel sufficiently competent to deliver brief interventions to patients

- Strongly Disagree
- Disagree
- Neither Agree nor Disagree
- Agree
- Strongly Agree

1. I think it is important that cardiology patients are asked about their alcohol habits

- Strongly Disagree
- Disagree
- Neither Agree nor Disagree
- Agree
- Strongly Agree

**Talking about lifestyle habits with patients**

Indicate how much you agree or disagree with the following statement (consider how sensitive these topics are in clinical conversations):

1. I feel comfortable discussing alcohol habits with patients

- Strongly Disagree
- Disagree
- Neither Agree nor Disagree
- Agree
- Strongly Agree

1. I feel comfortable discussing physical activity habits with patients

- Strongly Disagree
- Disagree
- Neither Agree nor Disagree
- Agree
- Strongly Agree

1. I feel comfortable discussing dietary habits with patients

- Strongly Disagree
- Disagree
- Neither Agree nor Disagree
- Agree
- Strongly Agree

1. I feel comfortable discussing smoking with patients

- Strongly Disagree
- Disagree
- Neither Agree nor Disagree
- Agree
- Strongly Agree

**Reliability of self-reported lifestyle habits**

Indicate the extent to which you agree or disagree with the following statement

1. Patients’ self-reporting of alcohol habits is reliable

- Strongly Disagree
- Disagree
- Neither Agree nor Disagree
- Agree
- Strongly Agree

1. Patients’ self-reporting of physical activity is reliable

- Strongly Disagree
- Disagree
- Neither Agree nor Disagree
- Agree
- Strongly Agree

1. Patients’ self-reporting of dietary habits is reliable

- Strongly Disagree
- Disagree
- Neither Agree nor Disagree
- Agree
- Strongly Agree

1. Patients’ self-reporting of smoking is reliable

- Strongly Disagree
- Disagree
- Neither Agree nor Disagree
- Agree
- Strongly Agree

1. If you have any other comments, please leave them below:

**S3 Table: Survey responses rates, by study site**

We contacted cardiology managers at hospitals in 15 towns across Sweden. Cardiology managers in three towns did not respond to our emails. Response rates for the 12 participating regions are reported below:

|  |  |  |  |  |
| --- | --- | --- | --- | --- |
|  | Town, region | Hospital type | Received survey | Responded to survey |
|  | Gävle, Gävleborg | DGH | 84* | 38/84 (45.2%) |
|  | Falun, Dalarna | DGH | 103 | 39/103 (37.9%) |
|  | Stockholm, Stockholm | Uni | 569 | 202/569 (35.5%) |
|  | Umeå, Västerbotten | Uni | 79 | 21/79 (26.6%) |
|  | Luleå, Norrbotten | DGH | 24 | 14/24 (58.3%) |
|  | Gothenburg, Västra Götaland | Uni | 259 | 106/259 (40.9%) |
|  | Karlstad, Värmland | DGH | 111 | 21/111 (18.9%) |
|  | Västerås, Västmanland | DGH | 102 | 57/102 (55.9%) |
|  | Örebro, Örebro | Uni | 184 | 43/184 (23.4%) |
|  | Lund/Malmö, Skåne | Uni | 182 | 53/182 (29.1%) |
|  | Uppsala, Uppsala | Uni | 177 | 30/177 (16.9%) |
|  | Linköping, Östergötland | Uni | 272 | 68/272 (25%) |
|  | Total |  | 2146 | 692/2146 (32.2%) |

*Partial estimate (based on estimated 60 nurses/assistant nurses employed at cardiology unit)

**S4 Fig: Bubbleplot to illustrate Spearman’s rank order correlation coefficient for perceived importance of screening and initiating SBI**


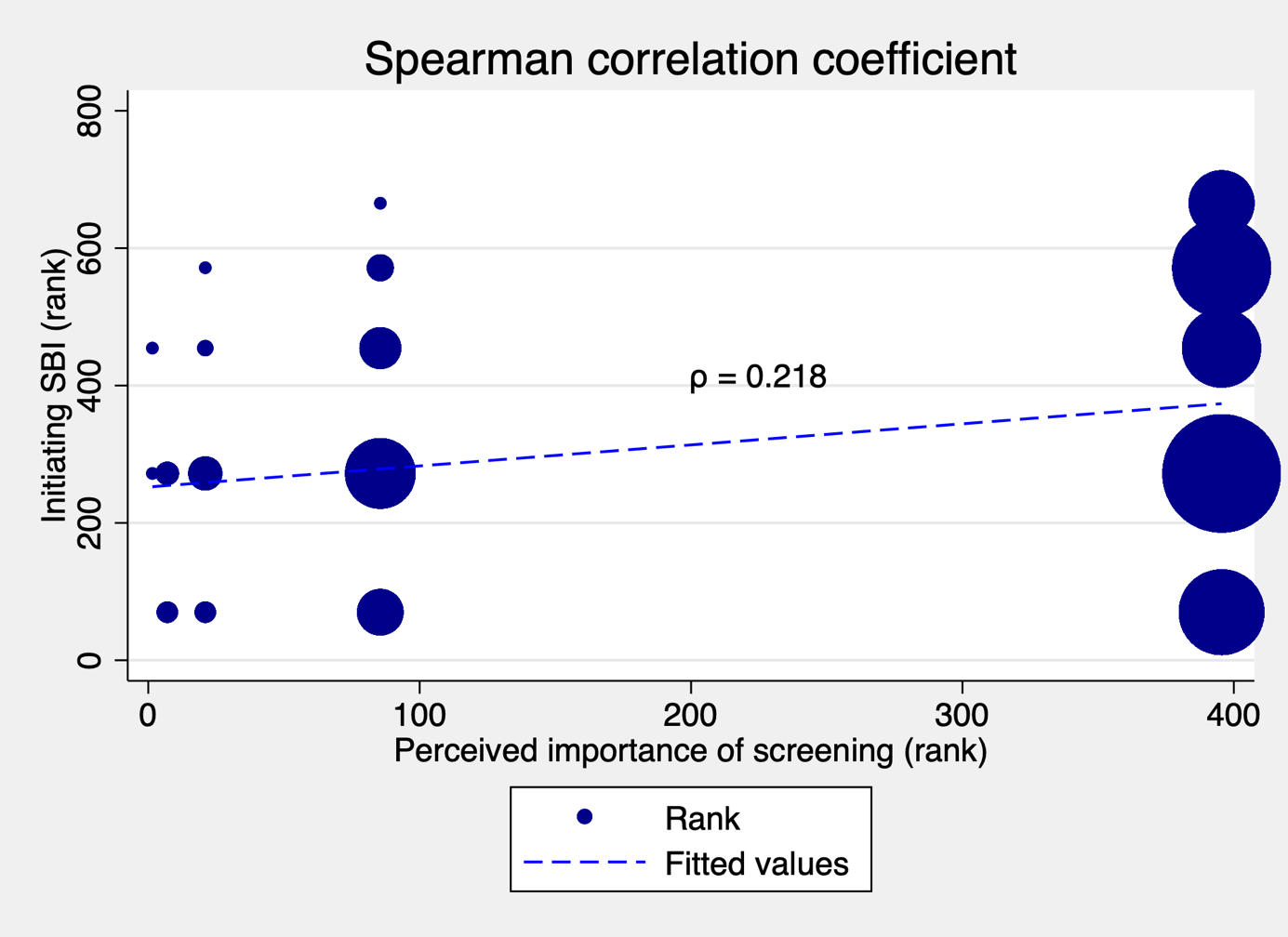


**
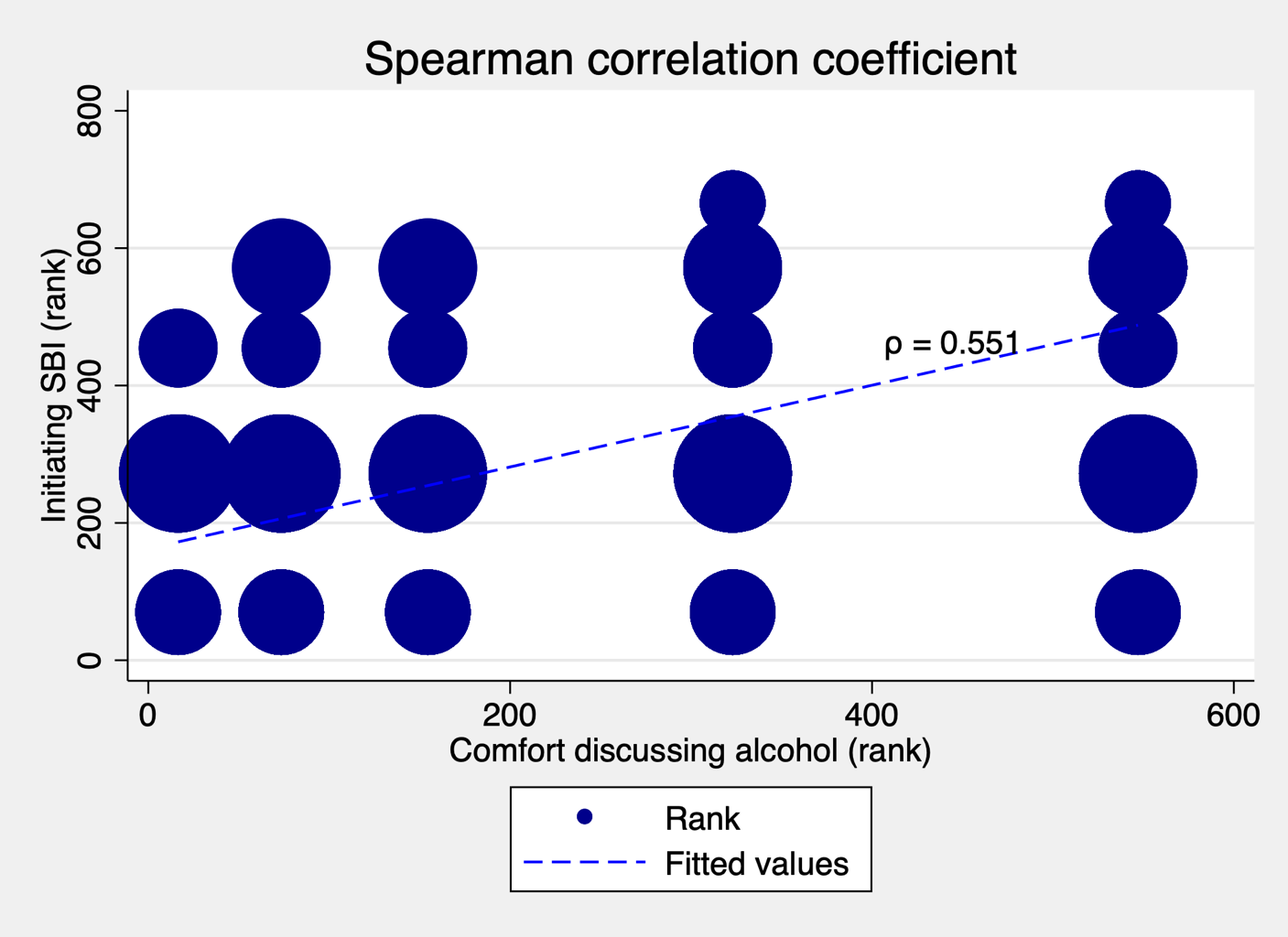
S5 Fig: Bubbleplot to illustrate Spearman’s rank order correlation coefficient for comfort discussing alcohol habits** **and initiating SBI**

**S6 Fig: Response categories for perceived reliability of self-reported alcohol habits and outcome variable, initiating SBI; frequencies and percentages (N=692)**

| **Perceived reliability of self-reported alcohol habits^a^** | | **Proportion, bar chart** | | **Initiating SBI** | |  |
| --- | --- | --- | --- | --- | --- | --- |
| *“Patients’ self-reporting of alcohol habits is reliable”* | Frequency (%) | 100% 0% 100% | | Frequency (%) | *“How often do you initiate conversations about alcohol use with your patients?”* | *Z*-value  (*p*-value)* |
| Strongly disagree | 32 (5.0) |  |  | 139 (20.1) | Never | *z* = 4.08  ***(p* < .001)** |
| Disagree | 257 (40.3) |  |  | 265 (38.3) | Rarely (with a few patients) |  |
| Neither agree nor disagree | 140 (21.9) |  |  | 100 (14.5) | Sometimes (about half of the time) |  |
| Agree | 200 (31.4) |  |  | 134 (19.4) | Often (with most patients) |  |
| Strongly agree | 9 (1.4) |  |  | 54 (7.8) | Always (with every patient) |  |

1. n=638

***Refers to Wilcoxon signed-rank test

**S7 Fig: Bubbleplot to illustrate Spearman’s rank order correlation coefficient for perceived reliability of self-reported alcohol habits and initiating SBI**

**
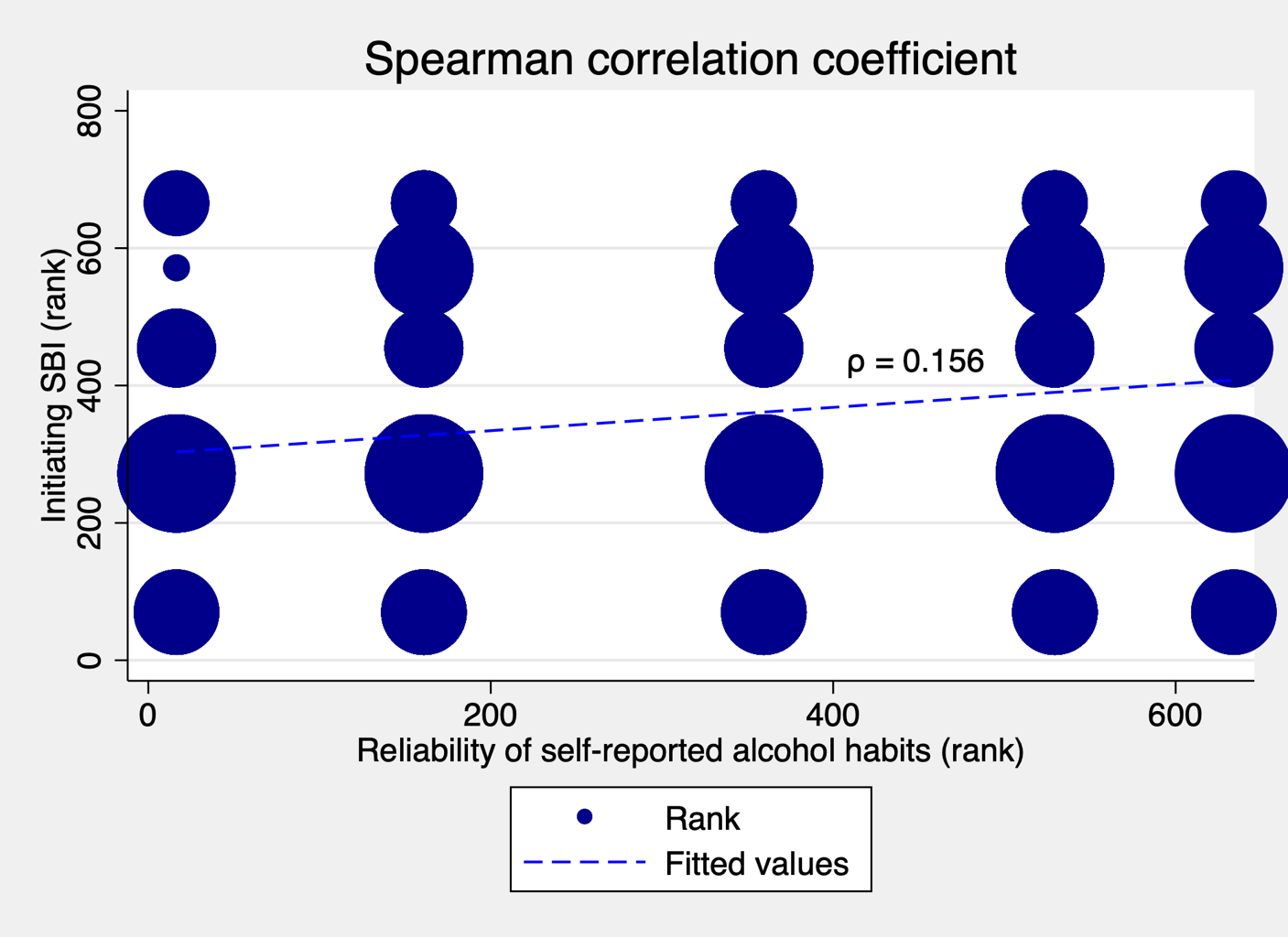
**

**S8 Fig: Response categories for perceived competence for screening and outcome variable, initiating SBI; frequencies and percentages (N=692)**

| **Perceived competence for screening^a^** | | **Proportion, bar chart** | | **Initiating SBI** | |  |
| --- | --- | --- | --- | --- | --- | --- |
| *“I feel sufficiently competent to ask patients about their alcohol habits”* | Frequency (%) | 100% 0% 100% | | Frequency (%) | *“How often do you initiate conversations about alcohol use with your patients?”* | *Z*-value  (*p*-value)* |
| Strongly disagree | 45 (6.9) |  |  | 139 (20.1) | Never | *z* = 16.81  ***(p* < .001)** |
| Disagree | 91 (14.0) |  |  | 265 (38.3) | Rarely (with a few patients) |  |
| Neither agree nor disagree | 98 (15.1) |  |  | 100 (14.5) | Sometimes (about half of the time) |  |
| Agree | 253 (38.9) |  |  | 134 (19.4) | Often (with most patients) |  |
| Strongly agree | 163 (25.1) |  |  | 54 (7.8) | Always (with every patient) |  |

1. n=650

***Refers to Wilcoxon signed-rank test

**S9 Fig: Response categories for perceived competence for brief interventions and outcome variable, initiating SBI; frequencies and percentages (N=692)**

| **Perceived competence for brief interventions^a^** | | **Proportion, bar chart** | | **Initiating SBI** | |  |
| --- | --- | --- | --- | --- | --- | --- |
| *“I feel sufficiently competent to deliver brief interventions to patients”* | Frequency (%) | 100% 0% 100% | | Frequency (%) | *“How often do you initiate conversations about alcohol use with your patients?”* | *Z*-value  (*p*-value)* |
| Strongly disagree | 117 (18.0) |  |  | 139 (20.1) | Never | *z* = 7.17  ***(p* < .001)** |
| Disagree | 138 (21.2) |  |  | 265 (38.3) | Rarely (with a few patients) |  |
| Neither agree nor disagree | 112 (17.2) |  |  | 100 (14.5) | Sometimes (about half of the time) |  |
| Agree | 217 (33.4) |  |  | 134 (19.4) | Often (with most patients) |  |
| Strongly agree | 66 (10.2) |  |  | 54 (7.8) | Always (with every patient) |  |

1. n=650

***Refers to Wilcoxon signed-rank test

**
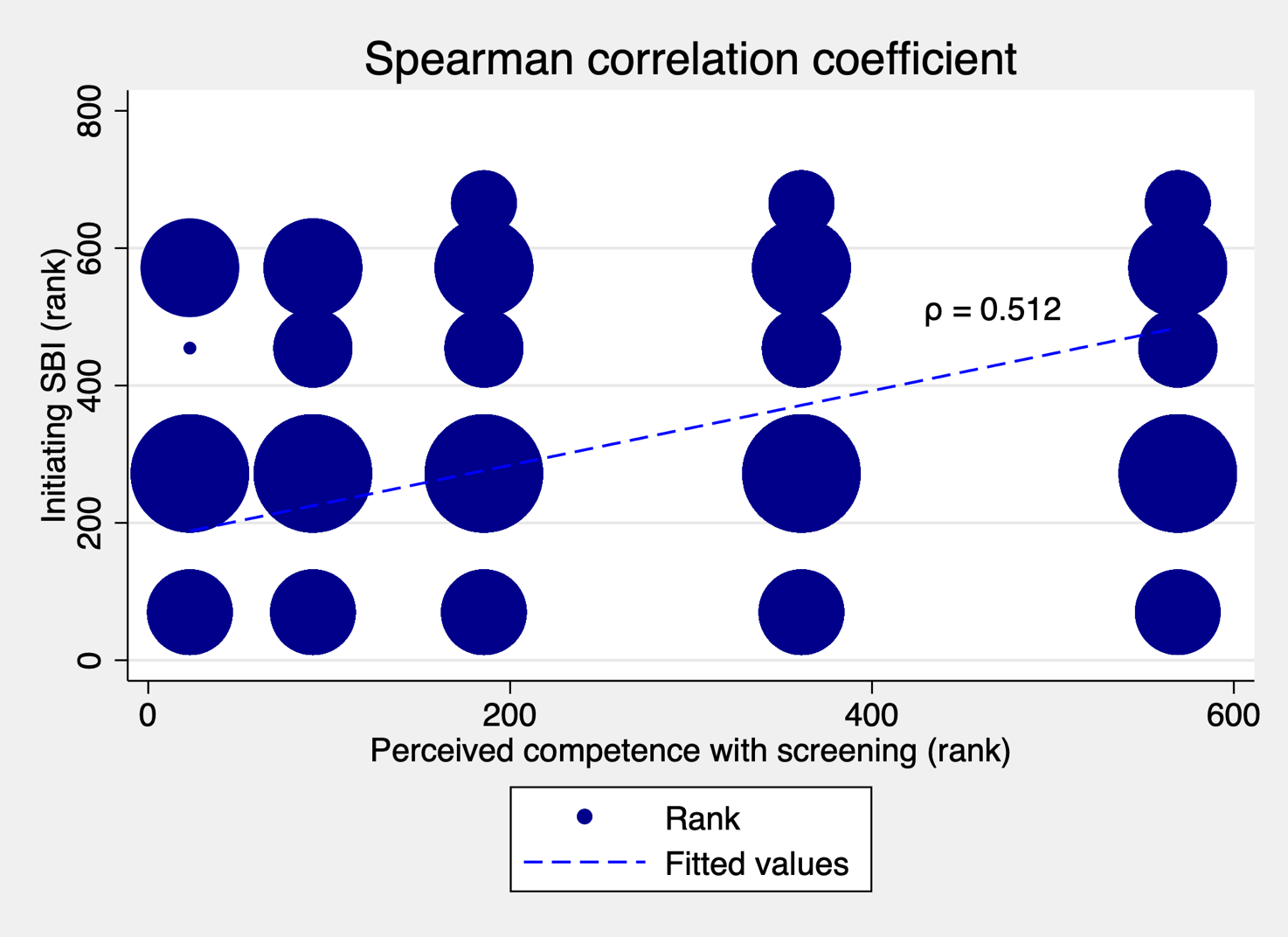
S10 Fig: Bubbleplot to illustrate Spearman’s rank order correlation coefficient for perceived competence for screening and initiating SBI**

**S11 Fig: Bubbleplot to illustrate Spearman’s rank order correlation coefficient for perceived
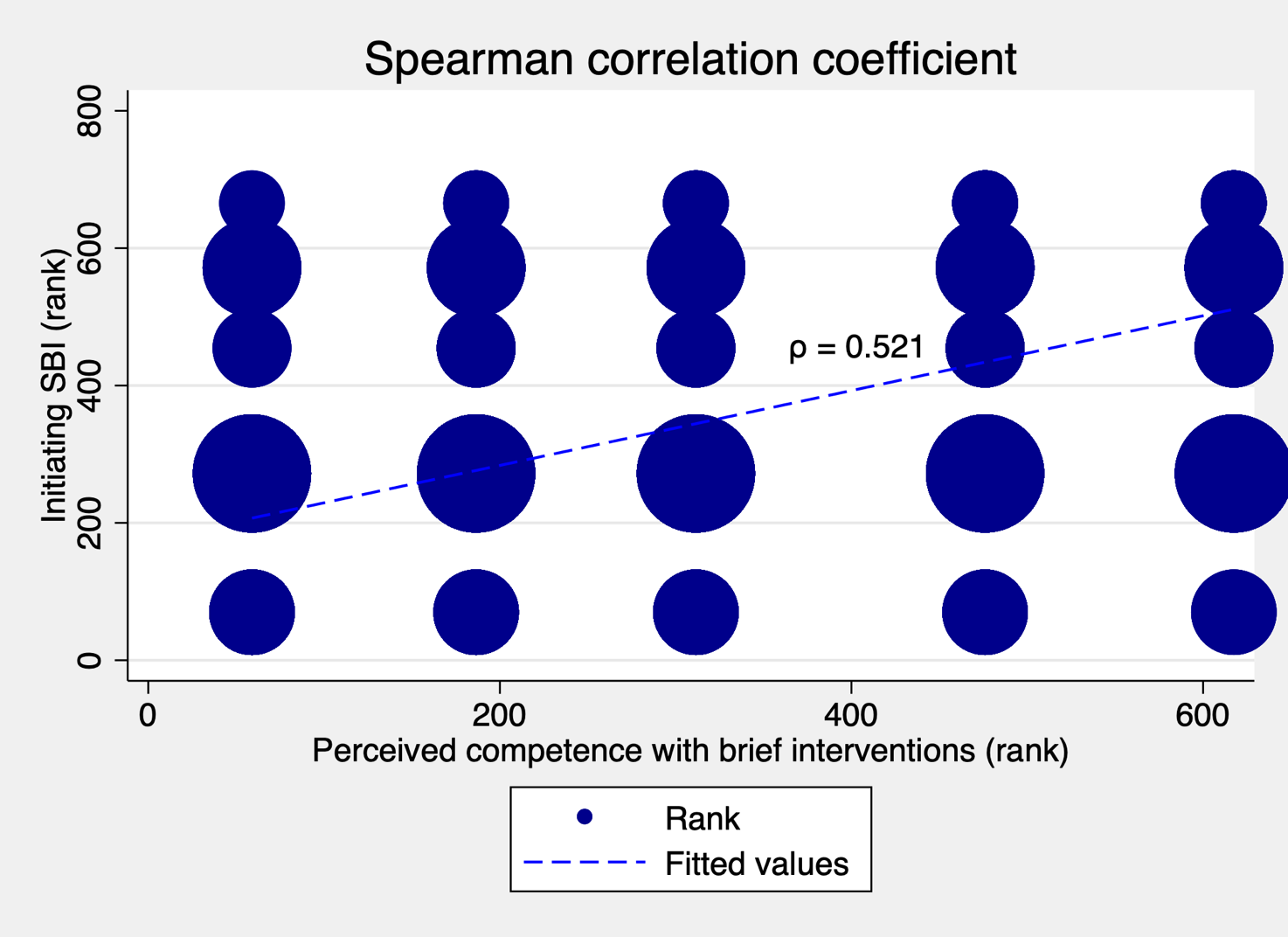
competence for brief interventions and initiating SBI**
